# Endoscopic discectomy of the herniated intervertebral disc and changes in quality-of-life EQ-5D-5L analysis

**DOI:** 10.1097/MD.0000000000034188

**Published:** 2023-06-30

**Authors:** Róbert Rapčan, Ladislav Kočan, Viktor Witkovsky, Simona Rapčanová, Juraj Mláka, Róbert Tirpák, Miroslav Burianek, Hana Kočanová, Janka Vašková, Miroslav Gajdoš

**Affiliations:** a Europainclinics, Bratislava, Slovak Republic; b Europainclinics, Poliklinika Terasa, Košice, Slovak Republic; c Europainclinics, Bardejov, Slovak Republic, Slovak Republic; d Clinic of Anaesthesiology and Intensive Care Medicine, East Slovak Institute of Cardiovascular Disease, Košice, Slovak Republic; e Institute of Measurement Science, Slovak Academy of Sciences, Bratislava, Slovak Republic; f Europainclinics, Prague, Czech Republic; g Clinic of Anaesthesiology and Intensive Care Medicine, Railway Hospital and Clinic Košice, Košice, Slovak Republic; h Department of Medical and Clinical Biochemistry, Faculty of Medicine, Pavol Jozef Šafárik University in Košice, Trieda SNP 1, Košice, Slovak Republic; i Department of Neurosurgery, Faculty of Medicine, Pavol Jozef Šafárik University in Košice, and Louis Pasteur University Hospital, Košice, Slovak Republic.

**Keywords:** back pain, discectomy, EQ-5D-5L questionnaire, leg pain, quality of life

## Abstract

Herniated lumbar discs are a common cause of low back pain, which can negatively impact the quality of life of working-age individuals. This study aimed to evaluate changes in the quality of life in patients with sciatica who underwent endoscopic discectomy, a minimally invasive surgical procedure. The study (ClinicalTrials.gov NCT02742311) included 470 patients who underwent transforaminal, interlaminar, or translaminar endoscopic discectomy. Quality of life and pain perception were evaluated by comparing statistically weighted values of EQ-5D-5L, EQ-VAS, Oswestry disability index, and numerical pain scales for lower limb and back pain before and 12 months after the endoscopic procedure. After the procedure, there was a significant improvement in the reduction of back and lower limb pain, as well as in all monitored questionnaires (*P* < .001), which persisted 12 months after the endoscopy. All evaluated dimensions of the EQ-5D-5L questionnaire indicated a significant improvement in the assessed quality of life (*P* < .001). The study showed that percutaneous endoscopic lumbar discectomy is an effective pain-treating intervention that can improve the quality of life. There was no observed difference in the percentage of complications or re-herniations when comparing the transforaminal and interlaminar, approaches.

## 1. Introduction

Low back pain with sciatica caused by lumbar disc herniation (LDH) affects nearly 60% of patients and is increasingly common as the global population ages. This condition is particularly prevalent in the working-age population, with 70% of disability caused by LDH occurring in people aged 20 to 65 years. The diagnosis of LHD is confirmed by magnetic resonance imaging, and the major symptoms include low back pain and sciatica. However, there is no clear evidence regarding the best treatment approach.^[1]^

Recently, percutaneous endoscopic lumbar discectomy (PELD) has been used as an alternative to open discectomy. PELD is a minimally invasive technique that reduces the use of general anesthesia and results in less damage to surrounding soft tissues and paravertebral musculature, faster wound healing time, reduced spine instability, and shorter hospitalization.^[2–4]^ The health-conditioned quality of life in patients with LHD is a result of both subjective perception of the disease and objective conditions.^[5–7]^ Therefore, various questionnaires are used to evaluate the health-related quality of life (HRQoL) of patients undergoing treatment.^[8,9]^

This study aimed to assess changes in the quality of life in patients with sciatica who underwent endoscopic discectomy using the EuroQol 5-dimension questionnaire. The study also evaluated the Oswestry disability index (ODI), the numerical scale of quality of life, and numerical scales of leg pain and back pain. The statistical weight values for the studied countries were determined. The results showed significant improvements in all monitored parameters, including a reduction in back and lower limb pain, as well as improvements in HRQoL that persisted 12 months after the endoscopic procedure. PELD was found to be an effective intervention for treating pain, and no significant differences were observed in complications or re-herniations when comparing the different approaches.

## 2. Materials and methods

### 2.1. Patients

**Figure 1.**
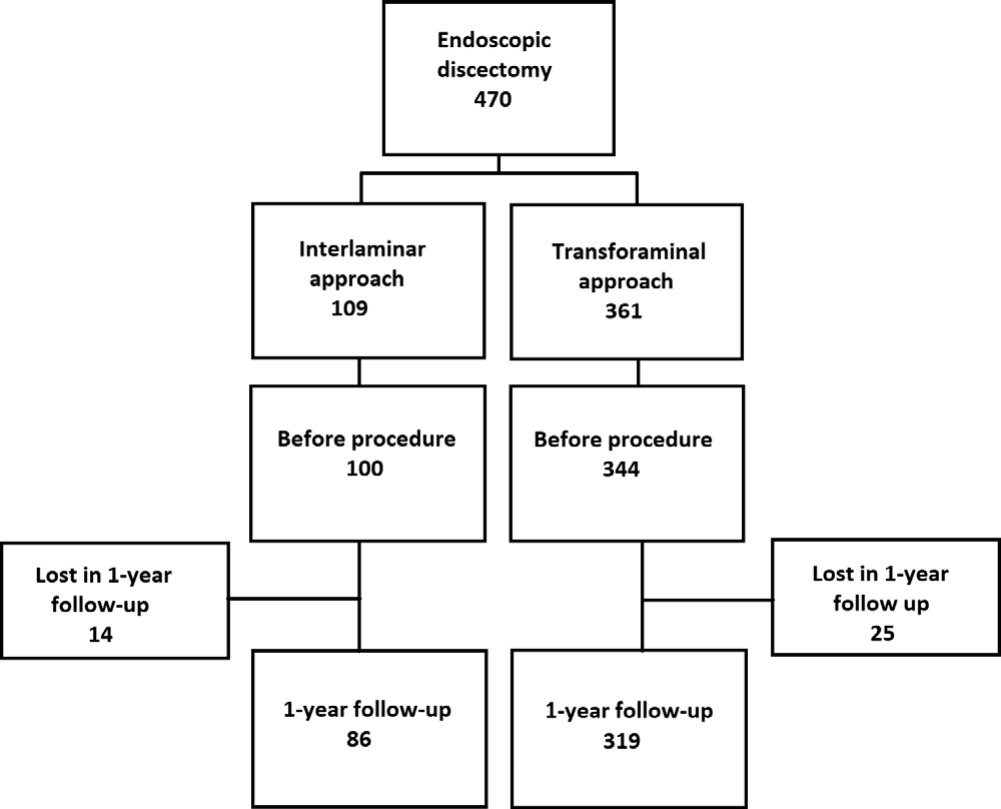
Flow chart of patient selection, enrollment, and follow-up in the study.

Between January 2016 and June 2021, we enrolled 470 patients who underwent ELD. These patients were between the ages of 18 to 80 years old and were classified according to their physical status using the American Society of Anesthesiology grading system (classes I, II, and III) (Fig. 1). The study was conducted in pain clinics located in Bratislava, Bardejov, Košice, Prague, and Brno in the Slovak and Czech Republic, and approved by a clinical investigation and recruitment process.

Inclusion criteria for the study were: being 18 years old or older, having signed informed consent, presenting with magnetic resonance imaging evidence of intervertebral disc herniation or sequestration, and experiencing permanent pain radiating to the lower limbs despite prior epidural steroid injection or similar interventions in conjunction with conservative treatment (such as rehabilitation and pharmacological treatment).

Exclusion criteria were: bleeding diathesis, urination or defecation problems, presence of infection or neoplasm, possible pregnancy, and patient disapproval. The procedure was performed by either a spine surgeon or an interventional pain management specialist, and each patient received a unique clinical trial ID number.

The primary outcomes of the study were the intensity of pain radiating to the back and legs, measured using the numerical rating scale (NRS) (ranging from 0 to 10), and the evaluation of the ODI, which assesses the extent of low back pain-related disability on a scale of 0 to 100%. Scores between 0 and 20% indicate minimal disability, while scores between 81 and 100% indicate bedridden patients or those with exaggerated symptoms. We also assessed the EuroQol survey using the EQ-5D-5L questionnaire. The descriptive system consists of five dimensions: mobility, self-care, usual activities, pain/discomfort, and anxiety/depression, with scores ranging from 1 to 5 (representing the best to worst state). The EuroQol survey also includes the EQ-VAS vertical visual analog scale, which utilizes a grading scale from 0 to 100 to evaluate the quality of life. A score of 0 indicates “The worst health you can imagine,” while a score of 100 indicates “The best health you can imagine.”

No significant changes were made to the methods after the study commenced. Prior to the operation, the NRS for back and leg pain, EQ-VAS, and ODI were assessed in one of the pain clinics participating in the study. The second assessment was conducted 12 months after the procedure, either during an appointment in one of the included centers or via a phone call follow-up, to assess pain severity by NRS and neurological state.

### 2.2. Surgery

#### 2.2.1. Transforaminal endoscopic discectomy (TFED).

This technique uses a natural entrance into the spinal canal (neuroforamen). The working channel for the endoscope is inserted through the paravertebral musculature latero-medially. During TFED, it is not necessary to disrupt the dorsal border of the spinal canal (the skeletal lamina of the vertebra and the ligamentum flavum). The risk of postoperative fibrosis formation is, therefore, minimal.

#### 2.2.2. Interlaminar endoscopic discectomy (ILED).

With more central protrusions that are harder to reach or more voluminous sequesters, especially at the L5/S1 level, the interlaminar approach was used. The working channel with the endoscope was introduced in the midline, very similar to a microdiscectomy. To enter the spinal canal, only a small part of the ligamentum flavum needs to be removed. The dural sac is then moved medially with a special maneuver to enable working in the central part of the channel.

#### 2.2.3. Translaminar endoscopic discectomy.

In cases, where entry into the spinal canal slightly above or below the interlaminar windows was desirable, translaminar endoscopic discectomy was used. The working channel for the affected disc level was inserted at the lateral inferior part of the lamina. Part of the lamina and ligamentum flavum are removed. This approach is very similar to ILED.

### 2.3. Anesthesia

The perioperative management of patients in our study involved several components. Prior to the operation, patients were premedicated with midazolam in a peroral dose ranging from 4 to 7.5 mg. During the procedure, standard monitoring was conducted, including electrocardiography, monitoring of oxygen saturation, and noninvasive blood pressure measurements.

During the procedures, the patients were positioned in the prone position. We employed the propofol-remifentanil target-controlled infusion sedation technique. Throughout the sedation process, the patients maintained consciousness and were responsive to our inquiries. They breathed spontaneously with the assistance of oxygen masks. The target-controlled infusion sedation technique was maintained by administering a continuous infusion of remifentanil at a rate ranging from 0.05 to 2 µg/kg/min. Additionally, propofol was administered with dosages varying from 0.5 to 4.0 μg/ml, depending on the specific needs of the surgical procedure.

During the operation, the surgical operator infiltrated the wound and operating canal with a mixture of local anesthetics. This combination consisted of 0.5% levobupivacaine and 1% trimecaine, with a total volume of up to 20 ml.

After the operation, patients were transferred to the recovery room where they were observed for up to 3 hours. During this time, standard monitoring was performed based on the patient’s clinical situation. This included continuous monitoring of vital signs such as heart rate, blood pressure, respiratory rate, and oxygen saturation. Additionally, any specific postoperative complications or concerns were addressed and managed accordingly.

To manage postoperative pain, patients received intravenous paracetamol at a dose of 1 g. For at-home medication, patients were prescribed tramadol at a dosage of 100 mg every 12 hours, diclofenac at a dosage of 75 mg per day, and paracetamol at a dosage of 500 mg every 8 hours.

These medications and interventions were part of our standardized protocol for perioperative management in our study. They were designed to ensure adequate pain control and patient comfort during the postoperative period.

### 2.4. Statistical methods

Statistical analysis of the EQ-5D-5L questionnaire scores (in each of the 5 dimensions considered) was conducted using Wilcoxon rank-sum test and, alternatively, the estimated reliability parameter (together with the associated confidence intervals [CIs]). The Wilcoxon rank-sum test is a standard statistical, nonparametric test that checks whether 2 independent samples come from continuous probability distributions (populations) with the same medians. Although the original version of the test was designed to compare continuous distributions, a simple generalization is possible for applications with discrete distributions (which is the case when we try to compare the distribution of questionnaire scores reflecting the degree of the symptom tested on a scale from 1 to 5). Secondly, as an alternative, we have used the estimated reliability parameter R=Prob(X<Y). For more details see Kotz et al,^[10]^ Zhou,^[11]^ and Rapčan et al.^[12]^

Descriptive statistical methods were also used to evaluate the results (mean, median, maximum, minimum, and SD). Examination of the distributional form for score and time data was determined by box plots. Each box plot indicated minimal value, lower quartile (lowest 25% of data), median, upper quartile (highest 25% of data), and maximal value. Normality of data distribution was assessed by the Shapiro–Wilk test. Homogeneity of variances was estimated using the Levene test. Differences between continuous variables were analyzed by a nonparametric Kruskal–Wallis 1-way test. A paired Student *t* test was used to assess the statistical significance of changes within each treatment group. *P* values of <0.05 were considered significant. Analyses were performed using MATLAB (R2021a) with Statistics and Machine Learning Toolbox version 12.1 (The MathWorks, Inc., MA) and the statistical software package SPSS version 11.0 (Chicago, IL) .

## 3. Results

Four hundred seventy patients were included in the statistical processing. Three hundred nineteen patients underwent TFED and 86 patients ILED with a 1-year follow-up (Fig. 1). Several parameters were evaluated (Table 1). Prior to discectomy, the patient's quality of life was assessed using an ODI questionnaire, a subsequent calculation of the ODI index, and a 5-dimensional EQ-5D-5L questionnaire was also assessed. The third questionnaire evaluating the quality of life from 0 to 100 was determined on the EQ-VAS scale. We also compared the intensity of back pain and leg pain measured on the numeric rating scale (0–10).

**Table 1:** Descriptive characteristics of the group of examined patients.

Surgical approach	Transforaminal	Interlaminar
	319	86
Gender M/F	140/179	660/26
Age	Min/Max/Med	Min/Max/Med
	21/79/45	18/74/51
Level of herniation		
L1/L2	0	0
L2/L3	3	0
L3/L4	18	0
L4/L5	169	0
L5/S1	122	86
L3/L4 + L4/L5	6	0
L4/L5 + L5/S1	1	0
Reoperations %	7%	6%

Comparing the parameters: ODI index, NRS, and EQ-VAS before the operation and 12 months after the operation showed a significant improvement in the reduction of back pain, painful lower limbs, as well as significant changes in the evaluated questionnaires *P* < .001 (Table 2). Before surgery, the median values of NRS for lower limb pain were 9, and NRS for back pain was 8.

**Table 2:** Evaluation of and Oswestry disability index, EQ-VAS, and pain intensity in the lower back and lower limbs according to the NRS in patients before surgery and 12 months after endoscopic discectomy.

Parameters	Time interval	Mean	SD	SEM	95% CI	*P* value
Oswestry disability index	Before procedure	54.11	20.71	1.186	36.71–41.38	<.001
	12 months follow-up	15.06				
EQ-VAS	Before procedure	39.13	30.32	1.506	−43.20 to −37.28	<.001
	12 months follow-up	79.38				
Numerical pain scale	Before procedure	7.83	3.32	0.919	4.69–5.44	<.001
Back pain	12 months follow-up	2.77				
Numerical pain scale	Before procedure	7.96	3.50	0.201	5.16–5.95	<.001
Leg pain	12 months follow-up	2.4				

CI = confidence interval, NRS = numerical rating scale, SD = standard deviation.

**Figure 2.**
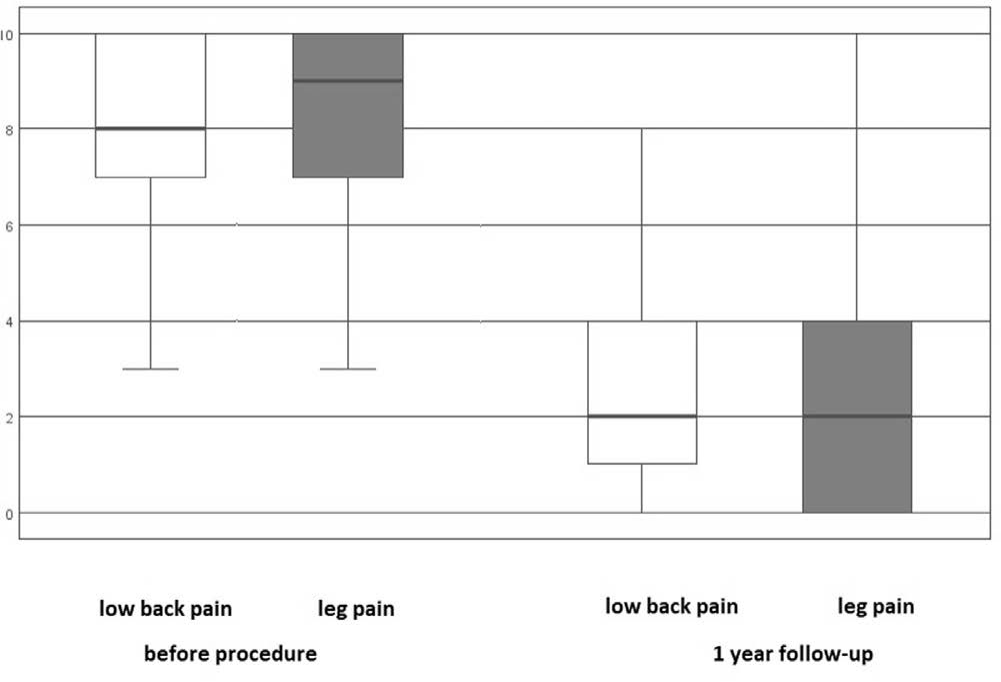
Evaluation of patients’ scores on the Numerical Pain Scale (NRS) for lower limb and back pain, before treatment, and 12 months after treatment.

**Figure 3.**
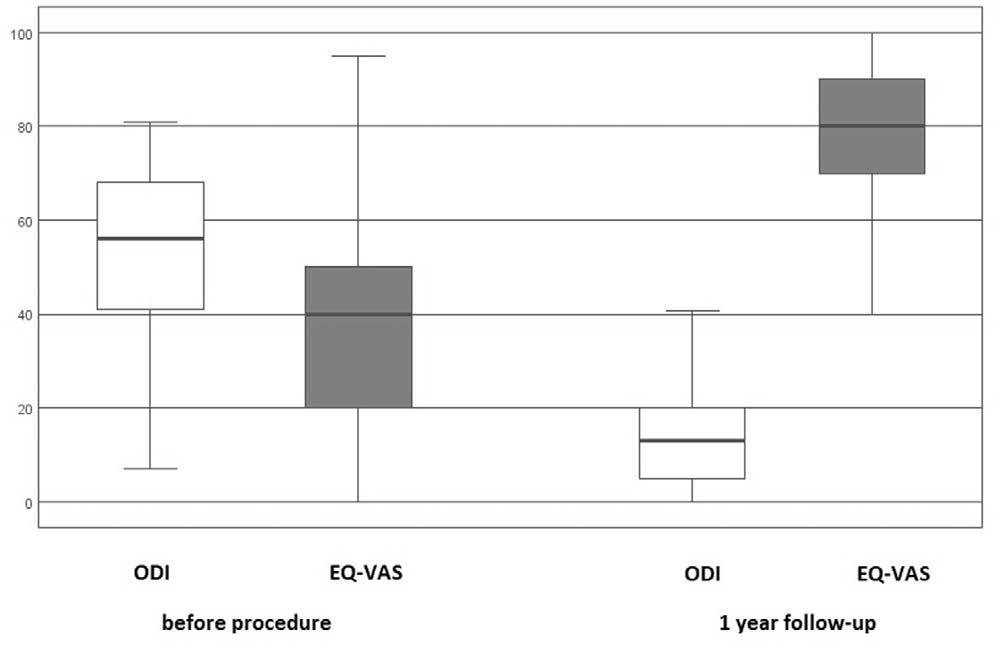
Evaluation of patients’ scores of Oswestry Disability Index (ODI) and overall health score (EQ-VAS) before treatment, and 12 months after treatment. Numerical scale (0–100) of ODI and EQ-VAS before the procedure and at the 1-year follow-up.

After 12 months, the median values for both groups in terms of back and leg pain dropped to 2, as shown in the box plots in Figure 2. The box plots represent the median, the box the 25th to 75th percentile, and the branches the range of the data, with a significant difference before and after the procedure. Furthermore, there was a statistically significant improvement in the quality of life, as indicated by the ODI and EQ-VAS questionnaires (Fig. 3), with *P* < .001. The responses of patients before the procedure and at the 1-year follow-up, according to the 5-dimensional EQ-5D-5L questionnaire, are summarized in Table 3 and Figure 3.

**Table 3:** The weights used for computing the weighted questionnaire value Q, which combines the information from all 5 dimensions of the EQ-5D-5L questionnaire.

Weight	Value	EQ-5D-5L dimension	Condition
w0	0.1279	All	Any level in questionnaire is 2 or 3
w0	0.2288	All	Any level in questionnaire is 4 or 5
w0	0.0000	All	Otherwise
w1	0.0659	Mobility	Mobility level is 2 or 3
w1	0.1829	Mobility	Mobility level is 4 or 5
w1	0.0000	Mobility	Otherwise
w2	0.1173	Self-care	Self-care level is 2 or 3
w2	0.1559	Self-care	Self-care level is 4 or 5
w2	0.0000	Self-care	Otherwise
w3	0.0264	Usual activities	Usual activities level is 2 or 3
w3	0.0860	Usual activities	Usual activities is 4 or 5
w3	0.0000	Usual activities	Otherwise
w4	0.0930	Pain/discomfort	Pain/discomfort level is 2 or 3
w4	0.1639	Pain/discomfort	Pain/discomfort level is 4 or 5
w4	0.0000	Pain/discomfort	Otherwise
w5	0.0891	Anxiety/depression	Anxiety/depression level is 2 or 3
w5	0.1290	Anxiety/depression	Anxiety/depression level is 4 or 5
w5	0.0000	Anxiety/depression	Otherwise

For each subject, the value Q was calculated by the formula $Q=1-w_{0}-w_{1}-w_{2}-w_{3}-w_{4}-w_{5}$, with the specified weights.

We formally evaluated the efficacy of endoscopic discectomy treatment in patients with sciatica. To do this, we tested statistical hypotheses about the equality of medians in the probability distributions of questionnaire responses in the patient groups against prespecified 1-sided alternative hypotheses. The alternative hypotheses stated that the distribution of response scores in the posttreatment group is stochastically smaller and has better quality-of-life measures than the distribution of response scores in the pretreatment group in terms of mobility, self-care, everyday activities, pain/discomfort, and anxiety/depression as defined in the 5-dimensional questionnaire EQ-5D-5L. Alternatively, we estimated the corresponding values of the reliability parameters along with the associated 2-sided 95% CIs. Alternatively, we estimated the corresponding values of the reliability parameters along with the associated 2-sided 95% CIs. As a summary measure, we also considered the combined (weighted) questionnaire responses using a carefully determined weighting. In particular, for each subject the combined value *Q* was calculated by the following formula: $Q=1-w_{0}-w_{1}-w_{2}-w_{3}-w_{4}-w_{5}$, with the weights specified in Table 3. For more details on the different weighing strategies and for discussion on selecting the specific weights see, for example, Rapčan et al,^[12]^ and Szende.^[13]^

Table 4 shows the calculated *P* values of the Wilcoxon rank-sum test and the estimated values of the reliability parameter *R* with the associated 2-sided 95% CI. These results are presented for all hypotheses about the equality of the probability distributions of the questionnaire responses in the patient groups considered for each of the 5 dimensions of the EQ-5D-5L questionnaire, that is mobility, self-care, everyday activities, pain/discomfort, anxiety/depression, and the combined weighted questionnaire score. The Wilcoxon rank-sum test convincingly rejected all considered null hypotheses of equality of the medians of the distributions of the questionnaire responses before and after treatment against the stated 1-sided alternative, which states that the respective dimension of the EQ-5D-5L questionnaire response is better after treatment than before treatment (After ˂ Before). Furthermore, the estimated values of the reliability parameters and the corresponding 95% CIs show that the calculated values for all considered EQ-5D-5L dimensions are higher than 0.5, which clearly confirms the results of the statistical tests.

**Table 4:** Calculated *P* values of the Wilcoxon rank-sum test and the estimated values of the reliability parameter *R*.

EQ-5D-5L dimension	Alternative hypothesis	Wilcoxon test *P* value	Reliability parameter *R* Prob(After<Before)	Lower	Upper
Mobility	After<Before	<.001	0.9112	0.8911	0.9298
Self-care	After<Before	<.001	0.9224	0.9031	0.9402
Usual activities	After<Before	<.001	0.9093	0.8881	0.9291
Pain/discomfort	After<Before	<.001	0.9320	0.9140	0.9486
Anxiety/depression	After<Before	<.001	0.6353	0.6051	0.6650
Weighted questionnaire	After<Before	<.001	0.9426	0.9258	0.9583

Calculated *P* values of the Wilcoxon rank-sum test and the estimated values of the reliability parameter *R* (shown together with the associated 2-sided 95% confidence interval) for 5 dimensions of the EQ-5D-5L questionnaire: mobility, self-care, usual activities, pain/discomfort, anxiety/depression, and also the combined weighted questionnaire result. These are used for testing the null hypothesis of equality of medians of the distributions of the questionnaire responses before and after treatment against the specified 1-sided alternative which specifies that the particular dimension of the EQ-5D-5L questionnaire response is better after the treatment than before the treatment (After < Before). The null hypothesis is rejected if the result of the Wilcoxon test is considered statistically significant (here at *P* < 0.05) or alternatively, if the reliability parameter *R* and all values included in the associated 95% confidence interval are above 0.5.

## 4. Discussion

This study aimed to evaluate clinical parameters, including pain intensity, ODI, and EQ-5D-5L scores, in patients with acute sciatica pain before and after endoscopic discectomy in the Slovak and Czech Republic. The study aimed to test the comprehensibility and face validity of EQ-5D-5L scores compared to other health measurement tools such as the ODI and NRSs for pain.

For all patients with LHD in the clinical trial, conservative therapy was initiated after the onset of clinical symptoms for 6 to 8 weeks. This included a combination of steroids, nonsteroidal anti-inflammatory drugs, physical therapy, epidural steroid injections, and rest. If the clinical state did not improve or worsened over time, the treatment strategy was changed to an endoscopic surgical approach. Although there are no proven differences in outcome between conservative and surgical treatment, timely neurosurgical treatment can lead to good long-term results. Nerve decompression performed at the right time is a good prognostic factor for neuronal recovery and can help prevent further irreversible neuronal damage and pain chronification.^[14,15]^

Among the monitored patients, the majority had a single-level herniated intervertebral disc in the intervertebral spaces L5/S1 (108 patients) and L4/L5 (169 patients). We observed a persistent improvement in the perception of back pain and pain radiating to the lower limbs, with a significant improvement in quality of life 1 year after the follow-up as measured by the EQ-VAS visual scale and the Oswestry disability questionnaire. These findings are consistent with the results of other clinical trials using various surgical approaches for lumbar herniated discs. A systematic review and meta-analysis of 99 articles focused on various spinal conditions found that spine surgery was associated with improved HRQoL for all groups in which postoperative scores were measured. The analysis included 22,312 cases for EQ-5D utilities, 2312 cases for SF-6D utilities, and 11,927 cases for SF-36 PCS scores with a median follow-up time of 12 months. Clinical trials monitoring the treatment of lumbar herniated discs with open surgical approaches, such as hemilaminectomy and microdiscectomy, have also shown the benefit of the procedures and improved patient quality of life after surgery. However, open surgery is associated with disadvantages such as extensive retraction and dissection of paraspinal muscles, longer operating time, larger wounds, and bone resection.^[10,16]^ Minimally invasive surgical approaches offer several advantages over open surgery, including the preservation of paraspinal structures and a reduction in postoperative pain, which often leads to earlier discharge. Additionally, these procedures can be performed under local anesthesia.^[16]^

A meta-analysis was conducted to compare the effectiveness of open lumbar surgery with lumbar endoscopic discectomy. The analysis included 9 randomized controlled trials with 1092 patients. The results showed that endoscopic surgery had slightly better clinical outcomes than open surgery based on the Macnab criteria, but this difference was not clinically significant. However, patients who underwent endoscopic surgery reported significantly higher satisfaction rates, lower intraoperative blood loss volume, and shorter hospital stays.^[6]^

Similarly, a meta-analysis compared the efficacy of PELD with other surgeries for LDH and demonstrated better outcomes of PELD in 14 clinical trials involving a total of 2528 patients. PELD had a shorter duration of surgery, lower blood loss, and similar complications compared to other surgeries for LDH. However, PELD operations resulted in a higher recurrence rate (relative risk = 1.65, 95% CI: 1.08–2.52; *P* = .021). There were no significant differences in quality of life and ODI before and after the procedure between the interventions. The study’s limitation was the relatively low number of clinical trials included.^[5]^ Nevertheless, published results concerning open-operative techniques are also associated with a good prognosis.

To evaluate the quality of life, we chose the EQ-5D-5L questionnaire as it offers a comprehensive assessment of a patient’s health status. This questionnaire measures 5 dimensions of perceived problems, each with 5 levels of severity, which improves sensitivity and reduces the ceiling effect. The EQ-VAS scale, which is easier to complete and score, was also used. The “Usual activities” dimension evaluates a patient’s performance in work, studying, housework, family, or leisure activities, as well as other dimensions like mobility, self-care, everyday activities, and anxiety/depression. Our analysis shows a significant improvement in the quality of life lasting >1 year after endoscopic surgery (*P* < .001). We also calculated the statistically weighted questionnaire values for each dimension of the EQ-5D-5L questionnaire in the Slovak and Czech region, which also indicate a significant improvement in social quality of life (*P* < .001).

Despite our study involving patients with a mean age of 46.3 years, the clinical improvement, reduction in lower limb pain, and improved quality of life after surgery are consistent with a 2-year follow-up study by Peng et al^[6]^ in younger patients with a mean age of 35.6 years.

An interesting finding from a prospective cohort study by Kapetanakis et al^[4]^ is that neither gender, muscle mass, nor body mass index has a significant effect on the final outcome of endoscopic discectomy.

A systematic analysis of clinical trials comparing the effectiveness of transforaminal and interlaminar endoscopic approaches in treating LHD, which reviewed 26 clinical trials involving 3294 patients, demonstrated a significant therapeutic benefit with both interventional approaches. However, the transforaminal approach was associated with higher efficacy, shorter operation time, and lower blood loss.^[7]^

In our clinical study, we selected the safest and most effective possible approach for each patient. For patients with higher levels of L3/4 and L4/5 herniations, we preferred the transforaminal approach. For herniations in the lower spinal segments L5/S1 due to anatomical conditions of the pelvis, we chose the interlaminar approach more often than the transforaminal approach. Comparing both approaches, we did not observe a higher percentage of complications or re-herniation.

We consider analgosedation with monitoring as a safe and beneficial method for endoscopic discectomy. It allows for effective communication with the patient, ensuring safety and minimizing the risk of nerve damage. Additionally, it promotes early recovery and a faster return to consciousness, while reducing the occurrence of postoperative nausea and vomiting. Despite potential challenges in airway access, we did not encounter any cases of acute respiratory failure requiring intubation.

### 4.1. Limitations

Our clinical trial on acute sciatica treatment had limitations that should be addressed. Firstly, using a prospective observational design instead of a randomized controlled trial introduced the potential for selection bias. The absence of a control group made it challenging to compare treatment effectiveness accurately. Including a control group would have enabled a more robust evaluation of endoscopic discectomy, microdiscectomy, and conservative treatments. The study involved 470 patients, but a broader age range (18–79 years) may have influenced the results. Additionally, a loss to follow-up of 39 patients resulted in missing data for statistical analysis, potentially impacting the robustness and generalizability of our findings. Future studies should minimize loss to follow-up and ensure comprehensive data collection. Recognizing these limitations is crucial for interpreting our findings accurately and planning future research in acute sciatica treatment.

## 5. Conclusions

Endoscopic discectomy is a highly effective minimally invasive surgical method for treating LHD, with a significant impact on the patient’s clinical state. Clinical assessments of the quality of life using different types of measurement instruments are useful in demonstrating the efficacy of pain-treating interventions.

## Author contributions

**Conceptualization:** Ladislav Kočan, Juraj Mláka, Róbert Tirpák.

**Data curation:** Róbert Rapčan, Ladislav Kočan, Viktor Witkovsky.

**Formal analysis:** Róbert Rapčan, Ladislav Kočan, Viktor Witkovsky, Miroslav Gajdoš.

**Funding acquisition:** Ladislav Kočan, Simona Rapčanová, Juraj Mláka.

**Investigation:** Ladislav Kočan, Viktor Witkovsky, Simona Rapčanová, Juraj Mláka, Róbert Tirpák, Hana Kočanová.

**Methodology:** Ladislav Kočan, Hana Kočanová.

**Project administration:** Ladislav Kočan, Janka Vašková.

**Resources:** Ladislav Kočan, Miroslav Burianek, Janka Vašková.

**Software:** Róbert Rapčan.

**Supervision:** Róbert Rapčan, Ladislav Kočan, Juraj Mláka.

**Validation:** Róbert Rapčan, Ladislav Kočan, Miroslav Burianek.

**Visualization:** Miroslav Burianek.

**Writing – original draft:** Ladislav Kočan, Simona Rapčanová, Róbert Tirpák, Hana Kočanová.

**Writing – review & editing:** Ladislav Kočan, Simona Rapčanová, Hana Kočanová.
